# Preparation of “Constrained Geometry” Titanium Complexes of [1,2]Azasilinane Framework for Ethylene/1-Octene Copolymerization

**DOI:** 10.3390/molecules22020258

**Published:** 2017-02-09

**Authors:** Seul Lee, Seung Soo Park, Jin Gu Kim, Chung Sol Kim, Bun Yeoul Lee

**Affiliations:** Department of Molecular Science and Technology, Ajou University, Suwon 443-749, Korea; dew6745@ajou.ac.kr (S.L.); zerg113@ajou.ac.kr (S.S.P.); juniori@ajou.ac.kr (J.G.K.); fubu50f@ajou.ac.kr (C.S.K.)

**Keywords:** olefin polymerization, titanium complex, constrained geometry, half-metallocene

## Abstract

The Me_2_Si-bridged *ansa*-Cp/amido half-metallocene, [Me_2_Si(η^5^-Me_4_C_5_)(N*^t^*Bu)]TiCl_2_, termed a “constrained-geometry catalyst (CGC)”, is a representative homogeneous Ziegler catalyst. CGC derivatives with the [1,2]azasilinane framework, in which the amide alkyl substituent is joined by the Si-bridge, were prepared, and the catalytic performances of these species was studied. Me_4_C_5_HSi(Me)(CH_2_CH=CH_2_)-NH(C(R)(R’)CH=CH_2_) (R, R’ = H or methyl; Me_4_C_5_H = tetramethylcyclopentadienyl) was susceptible to ring closure metathesis (RCM) when treated with Schrock’s Mo-catalyst to afford -Si(Me_4_C_5_H)(Me)CH_2_CH=CHC(R)(R’)NH- containing a six-membered ring framework. Using the precursors and the products of RCM, various CGC derivatives, i.e., [-Si(η^5^-Me_4_C_5_)(Me)CH_2_CH=CHC(R)(H)N-]TiMe_2_ (**13**, R = H; **15**, R = Me), [-Si(η^5^-Me_4_C_5_)(Me)CH_2_CH_2_CH_2_CH_2_N]TiMe_2_ (**14**), [(η^5^-Me_4_C_5_)Si(Me)(CH_2_CH=CH_2_)NCH_2_CH=CH_2_]TiMe_2_ (**16**), [(η^5^-Me_4_C_5_)Si (Me)(CH=CH_2_)NCH_2_CH=CH_2_]TiMe_2_ (**17**), and [(η^5^-Me_4_C_5_)Si(Me)(CH_2_CH_3_)NCH_2_CH_2_CH_3_]TiMe_2_ (**18**), were prepared. The catalytic activity of the newly prepared complexes was lower than that of CGC when activated with [Ph_3_C][B(C_6_F_5_)_4_]/iBu_3_Al. However, the catalytic activity of these species was improved by using tetrabutylaluminoxane ([iBu_2_Al]_2_O) instead of iBu_3_Al and the activity of **14/**[Ph_3_C][B(C_6_F_5_)_4_]/[iBu_2_Al]_2_O was comparable to that of CGC/[Ph_3_C][B(C_6_F_5_)_4_]/iBu_3_Al (4.7 and 5.0 × 10^6^ g/mol-Ti, respectively). Advantageously, the newly prepared complexes produced higher molecular weight poly(ethylene-*co*-1-octene)s than CGC.

## 1. Introduction

**Chart 1:**
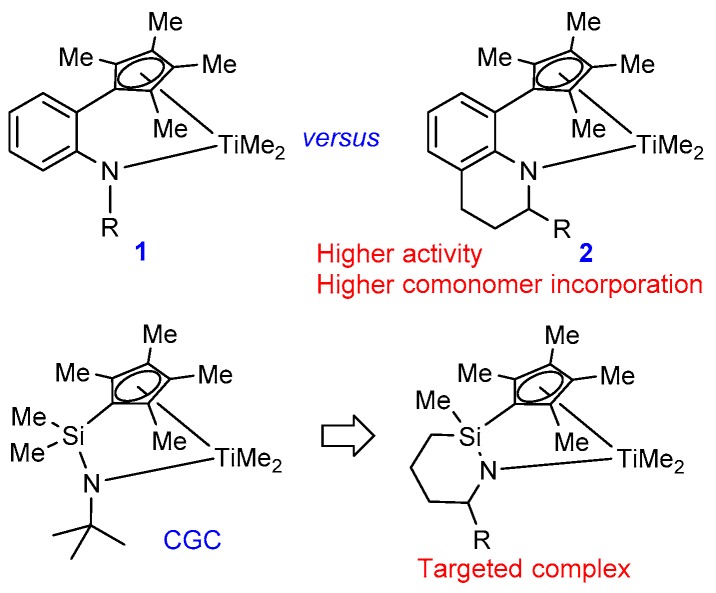
*Ortho*-phenylene-bridged half-metallocene complexes and the complexes targeted in this work.

Since the discovery of homogeneous metallocene catalysts (single-site polyolefin catalysts) in the 1970s by Kaminsky, their industrial impact has escalated, with current annual production of more than five million tons of polyolefins [1,2,3]. The initially developed family of metallocene-type catalytic species has expanded to include half-metallocenes constructed with a cyclopentadienyl (Cp) and an amido ligand [4,5,6,7,8,9,10], and further to post-metallocenes (or non-metallocenes) comprising non-cyclopentadienyl ligands [11,12,13,14,15,16]. Among the homogeneous Ziegler catalysts, Me_2_Si-bridged *ansa*-Cp/amido half-metallocenes, a typical example of which is [Me_2_Si(η^5^-Me_4_C_5_)(N*^t^*Bu)]TiCl_2_, are termed “constrained-geometry catalysts (CGCs, Chart 1)”, and have drawn significant attention from both industry and academia. CGC exhibits excellent catalytic performance, including high activity, high α-olefin incorporation, and high thermal stability [17,18,19,20,21,22]. We also reported a variety of *ansa*-Cp/amido half-metallocene congeners bearing the *ortho*-phenylene-bridge instead of the Me_2_Si-bridge (**1** in Chart 1) [23,24]. During the course of development of these catalysts, we observed that the tetrahydroquinoline (or tetrahydroquinaldine) derived species (**2** in Chart 1), in which the alkyl substituent in the amido ligand is joined to the *ortho*-phenylene-bridge, exhibited higher activity and higher α-olefin incorporation than the simple *ortho*-phenylene-bridged *ansa*-Cp/amido congeners **1** [25,26,27,28]. In fact, **2** displays excellent catalytic performance in ethylene/α-olefin copolymerization, thus enabling its use in commercial processes [29,30]. Joining the amide alkyl substituent and *ortho*-phenylene-bridge may afford a highly accessible reaction site for catalysis, resulting in higher activity and higher α-olefin incorporation. This observation prompted us to prepare CGC analogues in which the amide alkyl substituent is joined to the Si-bridge unit to form the [1,2]azasilinane framework (Chart 1). Various CGC derivatives have been reported; in most of these cases, the Me_4_C_5_-unit is replaced with other π-donor ligands or the N*^t^*Bu-unit is replaced with other amides or phosphides. However, the complexes targeted in this work have not previously been prepared [31,32,33,34,35,36,37,38,39].

## 2. Results

### 2.1. Synthesis and Characterization

**Scheme 1:**
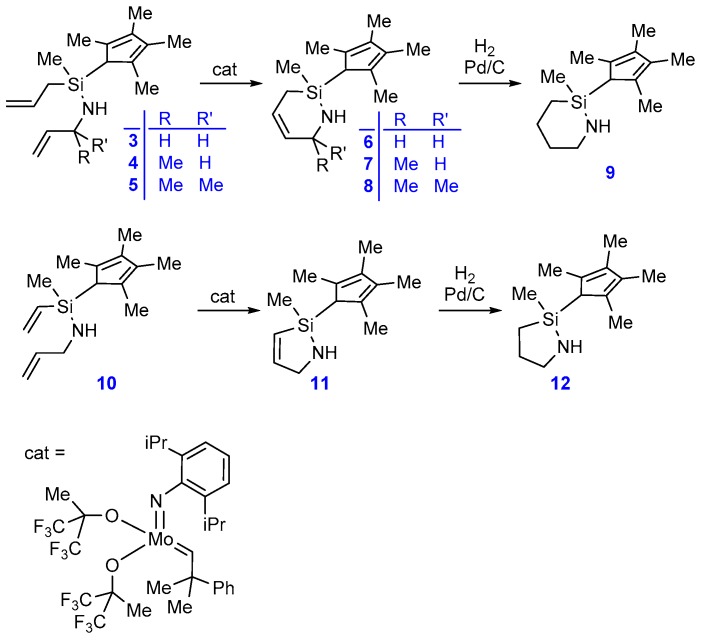
Construction of the ligand frameworks for the targeted complexes.

**Figure 1:**
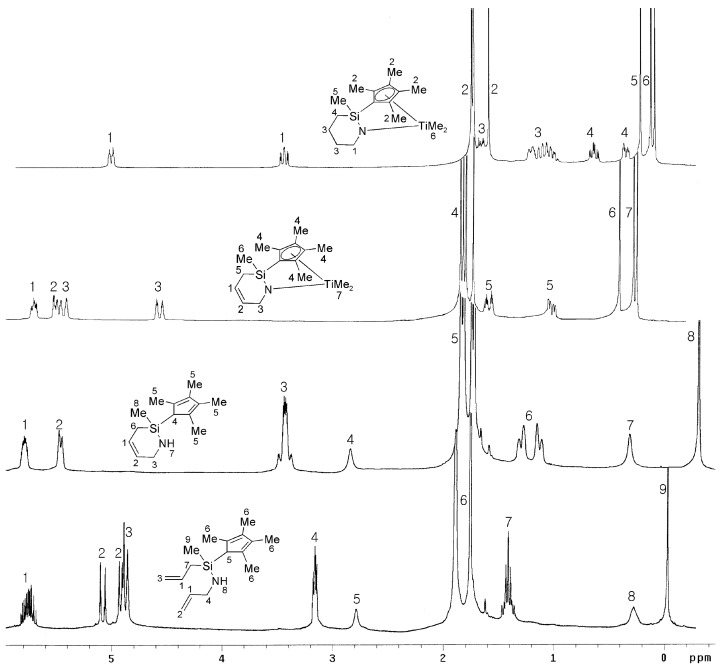
^1^H-NMR spectra of ring closure metathesis (RCM) precursor (**3**), RCM product (**6**), and titanium complexes (**13** and **14**).

The present strategy for construction of the [1,2]azasilinane framework involves the ring closure metathesis (RCM) of the two olefin units attached to the nitrogen and silicon atoms (Scheme 1). The precursor (**3**, Me_4_C_5_H-Si(Me)(CH_2_CH=CH_2_)-NH(CH_2_CH=CH_2_)) for RCM was easily prepared according to the scheme applied in the construction of the prototype CGC ligand by using commercially available chemicals, tetramethylcyclopentadiene (Me_4_C_5_H_2_), allyl(dichloro)methylsilane ((CH_2_=CHCH_2_)(Me)SiCl_2_), and allylamine. Initial RCM trials of **3** using Ru-catalysts (Grubbs 1st and 2nd generation catalysts and Hoveyda–Grubbs catalyst) for the synthesis of **6** were unsuccessful [40]. No ethylene gas was detected in the reaction monitored by ^1^H-NMR. Instead, migration of the double bond in the allylamine unit was observed. In contrast, the Mo-catalyst (Schrock catalyst, 2 mol %) cleanly converted **3** to the desired cyclic compound **6** [41,42,43,44]. When a closed-NMR tube with C_6_D_6_ was used, the signal of generated ethylene was observed at 5.24 ppm and the vinyl signals that were observed in the spectrum of **3** at 4.8–5.2 ppm and 5.7–5.9 ppm completely disappeared, while two new =CH signals were observed at 5.8 and 5.5 ppm (Figure 1). The RCM product **6** could be separated from the Mo-catalyst by vacuum distillation at 60 °C. The derivatives bearing methyl substituents at the α-position of the amino group (**7** and **8**) were also successfully prepared by RCM of the corresponding precursors **4** and **5**, which were prepared using 1-methylallylamine or 1,1-dimethylallylamine instead of allylamine. Hydrogenation of **6** using a Pd/C catalyst afforded **9** bearing the desired [1,2]azasilinane framework; the double bonds in the tetramethylcyclopentadiene units remained intact during the hydrogenation process. The characteristic vinyl signals in the ^1^H-NMR spectrum of **6** completely disappeared after hydrogenation. However, hydrogenation of the derivatives **7** and **8** bearing methyl substituent(s) at the α-position of the amino group was unsuccessful; the substrates remained intact under the identical hydrogenation conditions using the Pd/C catalyst. The five-membered analogue **11** was also successfully prepared via RCM of the vinylsilane derivative using the same Mo catalyst. Hydrogenation of **11** using the Pd/C catalyst was also successful to afford **12** bearing the [1,2]azasilolidine framework (Scheme 1).

**Chart 2:**
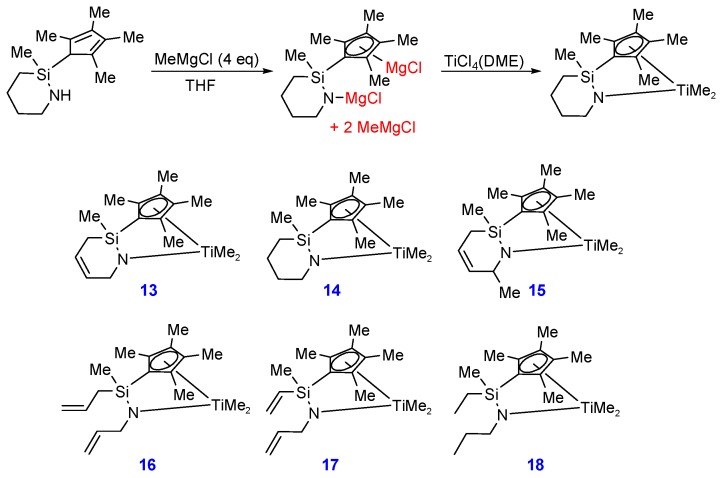
Prepared Ti-complexes.

The metalation of **6** using the methods introduced by Resconi et al. (treatment of 4 equiv. of MeLi in diethyl ether then with TiCl_4_) was unsuccessful [45,46]. The action of MeLi on **6** did not afford the desired dilithium compound (MeC_5_(Li)-Si(Me)(CH_2_CH=CH_2_)-N(Li)(CH_2_CH=CH_2_)), but instead resulted in destruction of the ligand framework. However, treatment of **6** with 4 equiv. of MeMgCl for a rather long reaction time (three days) in tetrahydrofuran (THF) at room temperature cleanly generated the desired dianion (MeC_5_(MgCl)-Si(Me)(CH_2_CH=CH_2_)-N(MgCl)(CH_2_CH=CH_2_)). Addition of TiCl_4_(DME) to the resulting solution containing the dianionic species and remaining 2 equiv. of MeMgCl afforded the desired complex **13** (Chart 2) in moderate yield (63%). The ^1^H-NMR spectrum showed Ti-Me and Si-Me signals at 0.59, 0.46, and 0.43 ppm as singlets, while C_5_Me_4_-signals were observed at 1.98, 1.96, 1.94, and 1.87 ppm as singlets. The two protons on -NCH_2_- are diastereotopic to each other and their signals were widely separated at 5.47 and 4.63 ppm with a large geminal coupling constant (^2^*J* = 19.5 Hz). The two =CH signals were observed at 5.7 and 5.5 ppm as multiplets (Figure 1). Complex **14** was also successfully prepared from **9** (hydrogenated compound of **6**) by the same method under the same conditions (treatment with 4 equiv. of MeMgCl and subsequent addition of TiCl_4_(DME)). The ^1^H-NMR spectrum of **14** (Figure 1) shows signals of the two diastereotopic -NCH_2_- protons at 5.18 and 3.65 ppm, indicating a notable up-field shift relative to the corresponding chemical shifts of **13** (5.47 and 4.63 ppm). When **7** (RCM precursor bearing a methyl substituent at the α-position of the amino group) was treated with MeMgCl, deprotonation was sluggish. Treatment of 2 equiv. of *n*BuLi in diethyl ether cleanly precipitated the dilithium compound, which was subsequently treated with TiCl_4_(DME) and 2 equiv. of MeMgCl simultaneously to afford the desired titanium complex **15**. Having two chiral centers, **15** was obtained as a mixture of two diastereomers and the ^1^H- and ^13^C-NMR signals were rather complex. Titanium complexes **16** and **17** were successfully generated from the respective RCM precursors **3** and **10**. In these cases, treatment of *n*BuLi in diethyl ether cleanly precipitated the corresponding dilithium compounds, from which the desired complexes were generated in good yields (84% and 88%) by treatment with TiCl_4_(DME) and 2 equiv. of MeMgCl. The vinyl groups in **17** were hydrogenated using a Pd/C catalyst to obtain **18** ([Me(Et)Si(η^5^-Me_4_C_5_)(κ^1^-NPr)]TiMe_2_). All complexes, except for **14**, were oily in nature with high solubility in hexane, and resistant to recrystallization, but the crude products extracted using hexane were pure based on ^1^H- and ^13^C-NMR analyses. Complex **14** was crystalline, but the crystals were formed as thin needles that were unsuitable for X-ray crystallography.

**Scheme 2:**
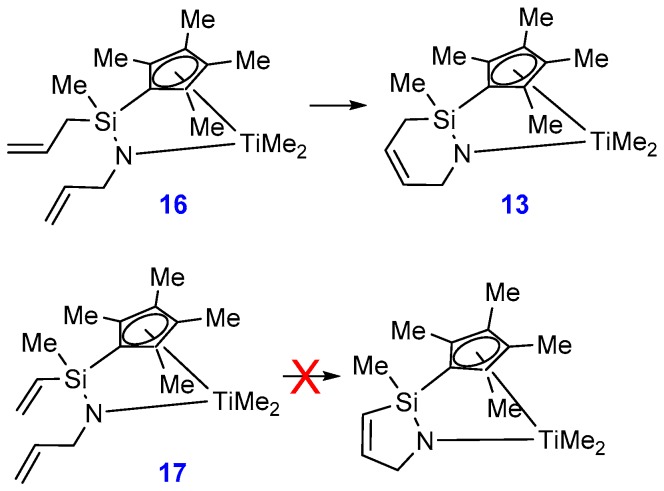
Ring closure metathesis (RCM) of the titanium complexes.

In the case of **8** (compounds bearing two methyl substituents at the α-position), treatment of *n*BuLi in diethyl ether cleanly precipitated the desired dilithium compound as a white solid, but metallation was unsuccessful. In the cases of **11** and **12** (five-membered ring compounds), various metallation attempts were also unsuccessful. In another route, RCM of the titanium complex **17** bearing vinyl-Si and allyl-N groups was also unsuccessfully attempted. In contrast, RCM of the titanium complex **16** bearing allyl-Si and allyl-N groups using the Mo-catalyst successfully generated six-membered ring complex **13** (Scheme 2). The bite angle in the cases of the five membered ring species **11** and **12** may not be practicable for chelation.

### 2.2. Polymerization Studies

The newly prepared complexes **13**–**18** along with the prototype CGC [Me_2_Si(η^5^-Me_4_C_5_)(N*^t^*Bu)]TiMe_2_ as a comparison were screened for ethylene/1-octene copolymerization in aliphatic hydrocarbon solvent (methylcyclohexane) after activation with [Ph_3_C][B(C_6_F_5_)_4_]. Triisobutylaluminum (iBu_3_Al) was used as a scavenger and the temperature was controlled in the range of 130–150 °C. The polymerization results are summarized in Table 1. None of the newly prepared complexes exhibited activity superior to that of CGC (entries 1–6 versus entry 7). The highest activity was observed with **15**, but the activity was ~2/3 that of CGC (3.1 × 10^6^ versus 5.0 × 10^6^ g/mol-Ti). The activities of **13** and **14** were ~1/3 that of CGC, while the activity of **18** was ~1/2 that of CGC. Complexes **16** and **17** containing acyclic olefin units showed low activity (<1/4 that of CGC).

**Figure 2:**
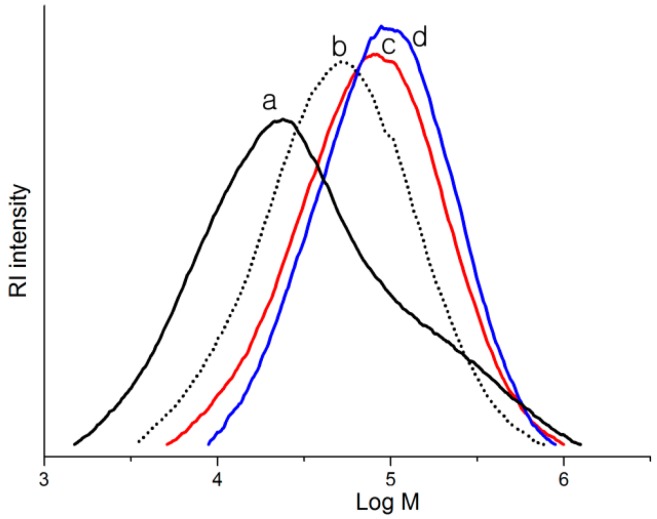
Gel permeation chromatography (GPC) curves of polymers generated with constrained-geometry catalyst (CGC)/[Ph_3_C][B(C_6_F_5_)_4_]/iBu_3_Al (**a**, entry 7), **14** (**b**), **13** (**c**), and **18** (**d**) activated with [Ph_3_C][B(C_6_F_5_)_4_]/[iBu_2_Al]_2_O.

Advantageously, the molecular weights of the products were higher than that of the polymer generated with CGC. Under the polymerization conditions employing methylcyclohexane at high temperature (120–150 °C), CGC produced relatively low-molecular-weight polymer (*M*_n_, 17 kDa) with a rather broad molecular weight distribution (*M*_w_/*M*_n_ 4.41), in contrast with the narrow molecular weight distributions (*M*_w_/*M*_n_ 1.7–3.4) obtained when the polymerization was performed in toluene at lower temperature (70–90 °C) [25,27,47]. In this study, a bimodal molecular weight distribution was observed for CGC with the low molecular weight portion comprising a main modal and the high molecular weight portion being a shoulder (Figure 2). All other complexes (**13**–**18**) except for CGC produced polymers with a narrow molecular weight distribution (*M*_w_/*M*_n_ 2.0–2.3). Among complexes **13**–**18**, **15** (bearing a methyl substituent at the α-carbon of the amido ligand) had the highest activity and also exhibited the lowest molecular weight (*M*_n_, 43 kDa). Complexes **13**, **14** and **18** produced relatively high molecular weight polymers (*M*_n_, 50–70 kDa) with reasonably high activities. Complexes **16** and **17** produced the highest molecular weight polymers (*M*_n_, 110 kDa), although their activities were low. Because of its low molecular weight, the polymer generated by CGC was sticky, in contrast with other generated polymers.

It was postulated that the low activities might be attributed to facile formation of “Ti(μ-R)AlR_2_”-type heterobimetallic species due to the highly accessible reaction site. Such heterobimetallic species have been recognized to be generated as a dormant state in the olefin polymerization process [48,49]. In order to suppress formation of such dormant species, Busico and coworkers treated MAO with 2,6-di-tert-butylphenol with the aim to trap free Me_3_Al contained in MAO, and Soares and coworkers used highly bulky trioctylaluminum as a scavenger [50,51,52]. In this study, we explored the use of tetrabutylaluminoxane ([iBu_2_Al]_2_O) instead of iBu_3_Al as a scavenger. [iBu_2_Al]_2_O is a commercially available chemical that is easily synthesized from iBu_3_Al by treatment with water [53,54]. By employing [iBu_2_Al]_2_O as a scavenger instead of iBu_3_Al, the activity was improved in all cases (entries 1–7 versus 8–16). The improvement was most significant in the case of **14**, where the activity improved 2.7-fold to reach 4.7 × 10^6^ g/mol-Ti, which is comparable to that of CGC/[Ph_3_C][B(C_6_F_5_)_4_]/iBu_3_Al (5.0 × 10^6^ g/mol-Ti). In the other cases, the activity improved ~1.5-fold. Both **14** and CGC were deactivated during the course of high temperature polymerization; the yields were marginally increased from 2.36 g (entry 9) to 2.56 g and from 3.58 g (entry 15) to 3.76 g for **14** and CGC, respectively, by lengthening the reaction time from 3 min to 6 min.

The *M*_n_ values declined somewhat with the use of [iBu_2_Al]_2_O instead of iBu_3_Al, but the general trend of the molecular weight versus the catalyst structure was the same (Figure 2). In the cases of **14**–**18**, the *M*_n_ values decreased by a factor of ~0.65 upon replacement of the iBu_3_Al with [iBu_2_Al]_2_O, while the decrease was marginal in the case of **18**, which invariably produced high molecular weight polymers (*M*_n_, ~70 kDa). The most significant decline of the *M*_n_ value was observed in the case of CGC, resulting in the production of a low-molecular-weight sticky polymer (*M*_n_, 9.3 kDa) with bimodal molecular weight distribution.

**Figure 3:**
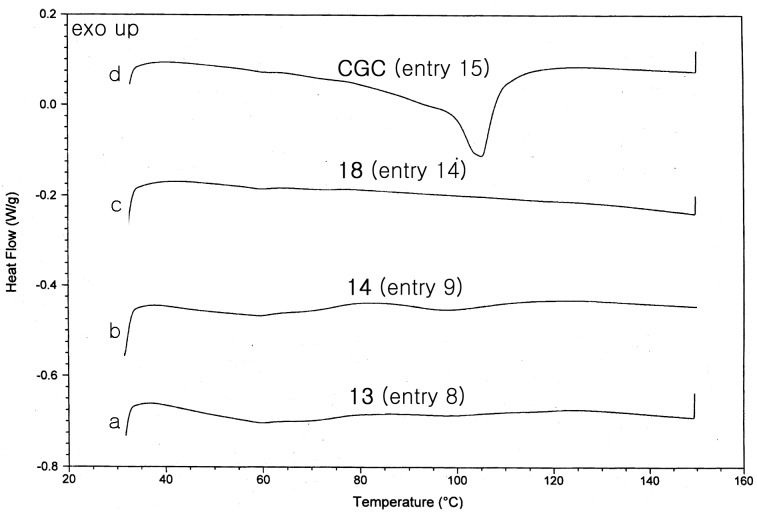
Differential scanning calorimeter (DSC) curves of the generated polymers.

Contrary to the expectation, the 1-octene contents of the samples generated using **13**–**17** were lower (12 mol %–15 mol %) than that of CGC (21 mol %) (Entries 8–13 versus 15). In the case of **18**, the 1-octene content was as high as that of CGC (19 mol % and 21 mol %, respectively). Strangely, whereas the melting signals observed in the differential scanning calorimeter (DSC) thermogram of the samples generated with **13**–**18** were extremely weak, the melting signal was observed around 100 °C for the sample generated with CGC, although the 1-octene content was even higher (Figure 3). This observation and the bimodal molecular weight distribution together indicate that the comonomer composition may not be uniform for the sample generated with the prototype CGC under the present polymerization conditions. When the polymerization temperature was lowered (80–120 °C), a unimodal distribution was observed in the GCP curve and the 1-octene content was significantly higher (36 mol %, entry 16). The melting signal was neither observed at DSC thermogram. These observations indicate that the prototype CGC generates another type of active species at high temperature, which produce high-molecular-weight polymer chains with less 1-octene content.

## 3. Experimental Section

### 3.1 General Remarks

All manipulations were performed under an inert atmosphere using standard glovebox and Schlenk techniques. Toluene, hexane, THF and diethyl ether were distilled from benzophenone ketyl. Methylcyclohexane was purified using a Na-K Alloy. NMR spectra were recorded on a Varian Mercury plus 400 instrument (Varian, Inc., Palo Alto, CA, USA). Mass spectra were obtained with a Micromass VG Autospec apparatus. Gel permeation chromatograms (GPC) were obtained at 140 °C in trichlorobenzene using a Waters Model 150-C+ GPC instrument (Waters, Milford, MA, USA) and the data were analyzed using a polystyrene analysis curve. 1-Methylallylamine and 1,1-dimethylallylamine were prepared by the procedure reported in the literature [55,56].

### 3.2. Compound ***3***

Allyl(dichloro)methylsilane ((CH_2_=CHCH_2_)(Me)SiCl_2_) (1.63 g, 10.5 mmol) was added dropwise to a stirred solution of tetramethylcyclopentadienyllithium (0.897 g, 7.0 mmol) in THF (40 mL) at −78 °C. The solution was stirred overnight at room temperature. All the volatiles were removed under vacuum, and hexane (30 mL) was then added in a glove box. The insoluble fraction was removed by filtration with the aid of Celite. Solvent in the filtrate was removed under vacuum and the resulting crude oil was distilled under full vacuum at 60 °C to obtain (CH_2_=CHCH_2_)(Me)Si(C_5_Me_4_)Cl (1.49 g, 88%). ^1^H-NMR (C_6_D_6_): δ 5.76–5.65 (m, 1H, CH=), 4.95–4.87 (m, 2H, =CH_2_), 2.94 (b, 1H, C_5_H), 1.95 (s, 3H, CH_3_), 1.88 (s, 3H, CH_3_), 1.70 (s, 6H, CH_3_), 1.61 (dd, *J* = 13.6, 7.6 Hz, 1H, SiCH_2_), 1.51 (dd, *J* = 13.6, 7.6 Hz, 1H, SiCH_2_), 0.19 (s, 3H, SiCH_3_) ppm. ^13^C{^1^H}-NMR (C_6_D_6_): δ 128.04, 137.78, 132.50, 131.46, 130.94, 115.26, 55.52, 23.62, 14.63, 14.60, 11.47, 11.41, −0.84 ppm. High resolution mass (HRMS) (EI): *m/z* calcd. ([M]^+^ C_13_H_21_ClSi) 240.1099. Found: 240.1101. (CH_2_=CHCH_2_)(Me)Si(C_5_Me_4_)Cl (1.49 g, 6.50 mmol) dissolved in THF (15 mL) was added dropwise to a stirred solution of LiN(H)CH_2_CH=CH_2_ (0.450 g, 7.15 mmol) in THF (25 mL) at −78 °C and the resulting solution was stirred overnight at room temperature. The volatiles were removed under vacuum and hexane (20 mL) was then added in a glove box. The insoluble fraction was removed by filtration with the aid of Celite. Solvent in the filtrate was removed under vacuum and the resulting crude oil was distilled under full vacuum at 70 °C (1.16 g, 73%). ^1^H-NMR (C_6_D_6_): 5.86–5.73 (m, 2H, CH=), 5.13 (ddd, *J* = 17.2, 3.6, 1.6 Hz, 1H, =CH_2_), 4.97 (ddd, *J* = 4.4, 3.6, 1.6 Hz, 1H, =CH_2_), 4.95–4.90 (m, 2H, =CH_2_), 3.25–3.20 (m, 2H, NCH_2_), 2.85 (b, 1H, C_5_H), 1.97 (s, 3H, CH_3_), 1.96 (s, 3H, CH_3_), 1.83 (s, 6H, CH_3_), 1.54–1.43 (m, 2H, SiCH_2_), 0.35 (b, 1H, NH), 0.05 (s, 3H, SiCH_3_) ppm. ^13^C{^1^H}-NMR (C_6_D_6_): δ 140.56, 135.78, 135.68, 135.18, 132.55, 132.25, 113.27, 112.81, 55.52, 44.92, 22.44, 14.78, 11.52, −3.96 ppm. HRMS (EI): *m/z* calcd ([M]^+^ C_16_H_27_NSi) 261.1913. Found: 261.1909.

### 3.3. Compound ***4***

1-Methylallylamine (0.950 g, 13.3 mmol) was added to (CH_2_=CHCH_2_)(Me)Si(C_5_Me_4_)Cl (1.27 g, 5.3 mmol) in THF (20 mL) and the resulting solution was stirred for 20 h. All volatiles were removed under vacuum and hexane (15 mL) was added. The generated HCl-allylamine salt was removed by filtration. Solvent in the filtrate was removed under vacuum to obtain colorless oil (1.38 g, 94%). The ^1^H-NMR spectrum indicated a mixture of two diastereomers in ca. 1:1 ratio. ^1^H-NMR (C_6_D_6_): δ 5.86–5.71 (m, 2H, CH=), 5.03 (ddt, *J =* 17.2, 12.0, 1.6 Hz, 1H, =CH_2_), 4.92–4.86 (m, 3H, =CH_2_), 3.36–3.29 (m, 1H, NCH), 2.84 (b, 1H, C_5_H), 1.97 (s, 3H, CH_3_), 1.95 (s, 3H, CH_3_), 1.81 (s, 6H, CH_3_), 1.49 (m, 2H, SiCH_2_), 1.04 and 1.02 (s, 3H, NCCH_3_), 0.31 and 0.28 (b, 1H, NH), 0.12 (s, 3H, SiCH_3_) ppm. ^13^C{^1^H}-NMR (C_6_D_6_): δ 145.83 and 145.76, 135.82, 135.68, 135.35 and 135.31, 132.71, 132.27, 113.28 and 113.25, 111.18 and 111.16, 55.69 and 55.59, 49.98 and 49.88, 25.80 and 25.72, 23.18 and 23.00, 15.06, 14.95, 11.54, −2.72 ppm. HRMS (EI): *m/z* calcd ([M]^+^ C_17_H_29_NSi) 275.2069. Found: 275.2071.

### 3.4. Compound ***5***

It was prepared by using the same procedure as utilized for **4** by using 1,1-dimethylallylamine (1.41 g, 91%). ^1^H-NMR (C_6_D_6_): δ 5.90 (dd, *J =* 17.2, 10.4 Hz, 1H, CH=), 5.84–5.77 (m, 1H, CH=), 5.04 (dd, *J =* 17.2, 1.4 Hz, 1H, =CH_2_), 4.96–4.90 (m, 2H, =CH_2_), 4.86 (dd, *J =* 10.4, 1.4 Hz, 1H, =CH_2_), 2.90 (s, 1H, C_5_H), 2.05 (s, 3H, CH_3_), 1.98 (s, 3H, CH_3_), 1.84 (s, 3H, CH_3_), 1.83 (s, 3H, CH_3_), 1.51 (d, *J =* 8.4 Hz, 2H, SiCH_2_), 1.15 (s, 3H, NC(CH_3_)_2_), 1.14 (s, 3H, NC(CH_3_)_2_), 0.56 (b, 1H, NH), 0.29 (s, 3H, SiCH_3_) ppm. ^13^C{^1^H}-NMR (C_6_D_6_): δ 149.41, 135.76, 135.65, 135.46, 133.30, 132.56, 113.28, 109.51, 55.97, 52.88, 31.65, 31.40, 24.12, 15.29, 15.06, 11.50, 0.86 ppm. HRMS (EI): *m/z* calcd ([M]^+^ C_18_H_31_NSi) 289.2226. Found: 289.2224.

### 3.5. Compound ***6***

Compound **3** (0.600 g, 2.29 mmol) in toluene (10 mL) was cooled to −30 °C and the Schrock catalyst (35 mg, 0.046 mmol) dissolved in toluene (1.0 mL) was then added. During the course of the reaction, ethylene gas was generated and was removed through a bubbler. After stirring for 3 h at room temperature, all volatiles were removed under vacuum. The resulting crude oil was distilled under full vacuum at 60 °C (0.507 g, 95%). ^1^H-NMR (C_6_D_6_): δ 5.88–5.82 (m, 1H, CH=), 5.54–5.51 (m, 1H, =CH), 3.58–3.46 (m, 2H, NCH_2_), 2.93 (b, 1H, C_5_H), 1.95 (s, 3H, CH_3_), 1.92 (s, 3H, CH_3_), 1.85 (s, 3H, CH_3_), 1.83 (s, 3H, CH_3_), 1.41 (d, *J =* 16.0 Hz, 1H, SiCH_2_), 1.25 (d, *J =* 16.0 Hz, 1H, SiCH_2_), 0.65 (b, 1H, NH), −0.18 (s, 3H, SiCH_3_) ppm. ^13^C{^1^H}-NMR (C_6_D_6_): δ 135.42, 132.19, 128.27, 123.66, 56.53, 43.31, 14.46, 11.89, 11.48, −4.33 ppm. HRMS (EI): *m/z* calcd ([M]^+^ C_14_H_23_NSi) 233.1600. Found: 233.1597.

### 3.6. Compound ***7***

It was prepared by using the same procedure applied to preparation of **6** by using **4**. The product was purified by distillation under full vacuum at 70 °C (0.900 g, 79%). The ^1^H-NMR spectrum indicated a mixture of two diastereomers in ca. 1:1 ratio. ^1^H-NMR (C_6_D_6_): δ 5.84–5.78 (m, 1H, =CH), 5.48–5.43 (m, 1H, CH=), 3.79–3.69 (m, 1H, NCH), 2.98 and 2.89 (b, 1H, C_5_H), 1.98 (s, 3H, CH_3_), 1.93 and 1.91 (s, 3H, CH_3_), 1.86 and 1.85 (s, 3H, CH_3_), 1.84 and 1.82 (s, 3H, CH_3_), 1.51 and 1.37 (dm, *J =* 17.0 Hz, 1H, SiCH_2_), 1.25 and 1.16 (dm, *J =* 17.0 Hz, 1H, SiCH_2_), 1.07 and 1.05 (d, *J =* 6.8 Hz, 3H, NCCH_3_), 0.57 and 0.53 (b, 1H, NH), −0.16 and −0.17 (s, 3H, SiCH_3_) ppm. ^13^C{^1^H}-NMR (C_6_D_6_): δ 135.57, 135.44, 135.37, 133.91 and 133.85, 132.19, 122.88 and 122.87, 56.74 and 56.51, 48.41 and 48.19, 27.41 and 27.36, 14.81, 14.69, 14.52, 12.06, 11.69, 11.51, −3.91 and −4.25 ppm. HRMS (EI): *m/z* calcd ([M]^+^ C_15_H_25_NSi) 247.1756. Found: 247.1755.

### 3.7. Compound ***8***

It was prepared by the same procedure as that used for **6** by using **5**. The product was purified by distillation under full vacuum at 80 °C (0.903 g, 70%). ^1^H-NMR (C_6_D_6_): δ 5.74 (dt, *J =* 10.4, 5.2 Hz, 1H, CH=), 5.45 (ddd, *J =* 10.4, 3.6, 1.6 Hz, 1H, =CH), 2.93 (b, 1H, C_5_H), 2.02 (s, 3H, CH_3_), 1.93 (s, 3H, CH_3_), 1.86 (s, 3H, CH_3_), 1.84 (s, 3H, CH_3_), 1.48 (ddd, *J =* 16.8, 5.2, 1.6 Hz, 1H, SiCH_2_), 1.20–1.15 (1H, SiCH_2_), 1.20 (s, 3H, NC(CH_3_)_2_), 1.15 (s, 3H, NC(CH_3_)_2_), 0.55 (b, 1H, NH), −0.17 (s, 3H, SiCH_3_) ppm. ^13^C{^1^H}-NMR (C_6_D_6_): δ 138.10, 135.60, 135.52, 132.16, 131.96, 121.34, 56.78, 52.26, 34.99, 34.79, 14.88, 14.73, 11.54, 11.51, −3.46 ppm. HRMS (EI): *m/z* calcd ([M]^+^ C_16_H_27_NSi) 261.1913. Found: 261.1914.

### 3.8. Compound ***9***

A bomb reactor was charged with **6** (0.500 g, 2.14 mmol) dissolved in THF (15 mL) and Pd/C (10 wt %, 0.20 g); H_2_ gas was introduced to a pressure of 10 bar. After stirring for 5 h, the remaining H_2_ gas was vented off. Pd/C was removed by filtration over Celite. Solvent in the filtrate was removed under vacuum and the resulting crude oil was pure based on ^1^H- and ^13^C-NMR analyses (0.502 g, 99%). ^1^H-NMR (C_6_D_6_): δ 3.00 (b, 1H, C_5_H), 2.98–2.91 (m, 1H, NCH_2_), 2.89–2.84 (m, 1H, NCH_2_), 1.96 (s, 6H, CH_3_), 1.86 (s, 3H, CH_3_), 1.84 (s, 3H, CH_3_), 1.75–1.70 (m, 2H, CH_2_), 1.49–1.43 (m, 2H, CH_2_), 0.85–0.78 (m, 1H, SiCH_2_), 0.64–0.57 (m, 1H, SiCH_2_), 0.53 (b, 1H, NH), −0.21 (s, 3H, SiCH_3_) ppm. ^13^C{^1^H}-NMR (C_6_D_6_): δ 135.42, 135.34, 132.25, 55.73, 43.82, 31.04, 23.77, 14.56, 14.49, 13.01, 11.49, −5.23 ppm. HRMS (EI): *m/z* calcd ([M]^+^ C_14_H_25_NSi) 235.1756. Found: 235.1758.

### 3.9. Compound ***10***

It was prepared by employing same procedure used for **3** by using dichloromethylvinylsilane instead of allyl(dichloro)methylsilane. The intermediate compound (CH_2_=CH)(Me)Si(C_5_Me_4_)Cl was purified by distillation under full vacuum at 50 °C (1.47 g, 83%). ^1^H-NMR (C_6_D_6_): δ 5.95 (dd, *J =* 19.4, 14.4 Hz, 1H, CH=), (dd, *J =* 14.4, 4.0 Hz, 1H, =CH_2_), (dd, *J =* 19.4, 4.0 Hz, 1H, =CH_2_), 2.97 (b, 1H, C_5_H), 1.98 (s, 3H, CH_3_), 1.92 (s, 3H, CH_3_), 1.73 (s, 6H, CH_3_), 0.17 (s, 3H, SiCH_3_) ppm. ^13^C{^1^H}-NMR (C_6_D_6_): δ 137.89, 134.56, 134.16, 131.38, 130.87, 55.93, 14.73, 14.59, 11.46, −2.09 ppm. HRMS (EI): *m/z* calcd ([M]^+^ C_12_H_19_ClSi) 226.0942. Found: 226.0945. Compound **10** was purified by distillation under full vacuum at 60 °C (1.16 g, 73%). ^1^H-NMR (C_6_D_6_): δ 6.10 (dd, *J =* 20.4, 14.6 Hz, 1H, CH=), 5.94 (dd, *J =* 14.6, 4.2 Hz, 1H, =CH_2_), 5.86–5.78 (m, 1H, CH=), 5.74 (dd, *J =* 20.4, 4.2 Hz, 1H, =CH_2_), 5.13 (ddd, *J =* 17.2, 3.6, 2.0 Hz, 1H, =CH_2_), 4.96 (ddd, *J =* 10.0, 3.6, 2.0 Hz, 1H, =CH_2_), 3.27–3.21 (m, 2H, NCH_2_), 2.86 (b, 1H, C_5_H), 2.00 (s, 3H, CH_3_), 1.98 (s, 3H, CH_3_), 1.84 (s, 6H, CH_3_), 0.32 (b, 1H, NH), 0.03 (s, 3H, SiCH_3_) ppm. ^13^C{^1^H}-NMR (C_6_D_6_): δ 140.66, 138.11, 135.68, 132.05, 112.80, 55.48, 44.97, 14.92, 11.56, −4.61 ppm. HRMS (EI): *m/z* calcd. ([M]^+^ C_15_H_25_NSi) 247.1756. Found: 247.1755.

### 3.10. Compound ***11***

It was prepared by employing the same procedure as that used for **6** by using **10**. The product was purified through distillation under full vacuum at 50 °C (1.56 g, 96%). ^1^H-NMR (C_6_D_6_): δ 6.77 (ddd, *J =* 10.0, 3.6, 1.6 Hz, 1H, =CH), 6.11 (dd, *J =* 10.0, 2.0 Hz, 1H, =CH), 3.71–3.62 (m, 2H, NCH_2_), 3.05 (b, 1H, C_5_H), 1.99 (s, 3H, CH_3_), 1.96 (s, 3H, CH_3_), 1.85 (s, 3H, CH_3_), 1.83 (s, 3H, CH_3_), 0.73 (b, 1H, NH), −0.16 (s, 3H, SiCH_3_) ppm. ^13^C{^1^H}-NMR (C_6_D_6_): δ 149.15, 135.79, 131.23, 126.55, 59.59, 52.28, 14.08, 14.05, 11.48, 11.44, –5.01 ppm. HRMS (EI): *m/z* calcd ([M]^+^ C_13_H_21_NSi) 219.1443. Found: 219.1441.

### 3.11. Compound ***12***

It was prepared by employing the same procedure as that used for **9** by using **11** (1.53 g, 97%). ^1^H-NMR (C_6_D_6_): δ 2.94–2.87 (m, 2H, NCH_2_), 1.96 (s, 3H, CH_3_), 1.94 (s, 3H, CH_3_), 1.86 (s, 3H, CH_3_), 1.84 (s, 3H, CH_3_), 1.81–1.53 (m, 2H, CH_2_), 0.96–0.60 (m, 2H, CH_2_), 0.66 (b, 1H, NH), –0.23 (s, 3H, SiCH_3_) ppm. ^13^C{^1^H}-NMR (C_6_D_6_): δ 135.56, 135.45, 131.95, 67.73, 57.33, 45.54, 25.77, 14.15, 14.07, 11.60, 11.46, –5.22 ppm.

### 3.12. Compound ***13***

Compound **6** (0.500 g, 2.14 mmol) dissolved in THF (40 mL) was added to a solution of MeMgCl in THF (3.0 M, 2.88 mL, 8.65 mmol). The resulting solution was stirred for three days. During the course of the reaction, methane gas evolved and was vented through a bubbler. TiCl_4_(DME) (0.600 g, 2.14 mmol) was added at –30 °C and the resulting solution was stirred at room temperature for 3 h. All volatiles were removed under vacuum, and hexane (10 mL) was added. The insoluble fraction was removed by filtration with the aid of Celite. Solvent in the filtrate was removed under vacuum. The resulting dark brown oil was redissolved in hexane (5 mL) and filtration was performed another time. A dark brown oil was obtained by removal of the solvent from the filtrate, which was pure based on ^1^H- and ^13^C-NMR analyses (0.437 g, 63%). ^1^H-NMR (C_6_D_6_): 5.77–5.70 (m, 1H, =CH), 5.57–5.52 (m, 1H, =CH), 5.47 (ddd, *J =* 19.5, 6.0, 3.2 Hz, 1H, NCH_2_), 4.63 (dm, *J =* 19.5 Hz, 1H, NCH_2_), 1.98 (s, 3H, CH_3_), 1.96 (s, 3H, CH_3_), 1.94 (s, 3H, CH_3_), 1.87 (s, 3H, CH_3_), 1.73 (dt, *J =* 17.2, 8.0 Hz, 1H, SiCH_2_), 1.18 (ddt, *J =* 17.2, 6.8, 1.2 Hz, 1H, SiCH_2_), 0.59 (s, 3H, SiCH_3_), 0.46 (s, 3H, TiCH_3_), 0.43 (s, 3H, TiCH_3_) ppm. ^13^C{^1^H}-NMR (C_6_D_6_): δ 134.67, 133.29, 130.26, 128.56, 127.26, 122.87, 95.54, 53.75, 52.11, 49.06, 15.24, 14.94, 14.02, 11.99, 11.89, 2.84 ppm.

### 3.13. Compound ***14***

It was prepared by employing the same procedure used for **13** by using **9**. A dark brown oil was obtained by removal of the solvent from the filtrate, which was pure based on ^1^H- and ^13^C-NMR analyses (0.403 g, 58%). ^1^H-NMR (C_6_D_6_): δ 5.20–5.14 (m, 1H, NCH_2_), 3.68–3.61 (m, 1H, NCH_2_), 2.01 (s, 3H, CH_3_), 1.99 (s, 6H, CH_3_), 1.94–1.88 (m, 1H, CH_2_), 1.87 (s, 3H, CH_3_), 1.49–1.44 (m, 1H, CH_2_), 1.41–1.29 (m, 2H, CH_2_), 0.94 (td, *J =* 14.0, 5.6 Hz, 1H, CH_2_), 0.68–0.62 (dm, *J =* 14.0 Hz, 1H, CH_2_), 0.57 (s, 3H, SiCH_3_), 0.47 (s, 3H, TiCH_3_), 0.41 (s, 3H, TiCH_3_) ppm. ^13^C{^1^H}-NMR (C_6_D_6_): δ 134.70, 132.92, 128.19, 127.02, 95.87, 53.44, 51.48, 48.41, 31.75, 23.09, 15.81, 15.22, 14.86, 12.02, 11.91, 0.82 ppm. Anal. Calcd. for C_16_H_29_NSiTi (311.36 g/mol): C 61.72, H 9.39, N 4.50%. Found: C 61.69, H 9.06, N 4.94%.

### 3.14. Compound ***15***

*n*BuLi (8.8 mmol) was added to **7** (1.16 g, 4.4 mmol) dissolved in diethyl ether (15 mL) at −30 °C and the resulting solution was stirred for 3 h at −30 °C. The dilithiated compound precipitated as a white solid, which was isolated by decantation and dried under vacuum (1.18 g, 98%). The isolated solids were dissolved in THF (30 mL), and MeMgCl in THF (3.0 M, 2.87 mL, 8.6 mmol) and TiCl_4_(DME) (1.21 g, 4.3 mmol) were successively added at −30 °C. After stirring for 3 h, the product was isolated by using the same procedure employed for **13** (0.713 g, 49%). The ^1^H-NMR spectrum indicated a mixture of two diastereomers in ca. 1:1 ratio. ^1^H-NMR (C_6_D_6_): δ 5.72–5.44 (m, 2H, CH=), 5.58 and 4.45 (m, 1H, NCH), 2.08 and 1.43 (d, *J =* 7.2 Hz, 3H, NCCH_3_), 1.99 and 1.98 (s, 3H, CH_3_), 1.97 and 1.96 (s, 3H, CH_3_), 1.93 and 1.87 (s, 3H, CH_3_), 1.90 (s, 3H, CH_3_), 1.66 (dm, *J =* 17.2, 3.6 Hz, 1H, SiCH_2_), 1.14 and 1.09 (dd, *J =* 17.2, 6.8 Hz, 1H, SiCH_2_), 0.58 and 0.54 (s, 3H, SiCH_3_), 0.46 and 0.45 (s, 3H, TiCH_3_), 0.43 and 0.41 (s, 3H, TiCH_3_) ppm. ^13^C{^1^H}-NMR (C_6_D_6_): δ 136.35 and 135.72, 134.59, 133.60, 133.53, 133.29, 128.87, 128.75, 128.52, 127.23, 122.41 and 121.94, 96.05 and 95.45, 57.79 and 57.20, 53.66 and 52.96, 50.18 and 48.85, 27.04 and 26.54, 15.28, 15.11, 14.97, 14.13, 13.56, 12.02, 11.97, 11.95, 4.46 and 1.41 ppm.

### 3.15. Compound ***16***

It was prepared by using the same procedure as that employed for **15** by using **3**. A dark brown oil was obtained by removal of the solvent from the filtrate, which was pure based on ^1^H- and ^13^C-NMR analyses (1.27 g, 84%). ^1^H-NMR (C_6_D_6_): 6.02–5.81 (m, 2H, CH=), 5.16–4.92 (m, 4H, =CH_2_), 4.42 (ddt, *J =* 15.6, 6.4, 1.6 Hz, 1H, NCH_2_), 4.26 (ddt, *J =* 15.6, 6.4, 1.6 Hz, 1H, NCH_2_), 1.96 (s, 6H, CH_3_), 1.89 (s, 3H, CH_3_), 1.85 (s, 3H, CH_3_), 0.51 (s, 3H, TiCH_3_), 0.49 (s, 3H, TiCH_3_), 0.41 (s, 3H, SiCH_3_) ppm. ^13^C{^1^H}-NMR (C_6_D_6_): δ 139.40, 134.40, 134.09, 133.73, 128.20, 114.58, 114.27, 95.97, 53.78, 51.39, 27.40, 15.21, 15.12, 12.01, 0.48 ppm.

### 3.16. Compound ***17***

It was prepared by using the same procedure as employed for **15** by using **10**. A dark brown oil was obtained by removal of the solvent from the filtrate, which was pure based on ^1^H- and ^13^C-NMR analyses (1.39 g, 88%). ^1^H-NMR (C_6_D_6_): δ 6.23 (dd, *J =* 20.0, 14.4 Hz, 1H, CH=), 6.01–5.96 (m, 1H, CH=), 5.92 (dd, *J =* 14.4, 3.6 Hz, 1H, =CH_2_), 5.72 (dd, *J =* 20.0, 3.6 Hz, 1H, =CH_2_), 5.16 (dm, *J =* 16.8 Hz, 1H, =CH_2_), 5.02 (dm, *J =* 9.2 Hz, 1H, =CH_2_), 4.86 (ddt, *J =* 15.2, 6.0, 1.6 Hz, 1H, NCH_2_), 4.59 (ddt, *J =* 15.2, 6.0, 1.6 Hz, 1H, NCH_2_), 1.98 (s, 3H, CH_3_), 1.97 (s, 3H, CH_3_), 1.96 (s, 3H, CH_3_), 1.90 (s, 3H, CH_3_), 0.55 (s, 3H, TiCH_3_), 0.53 (s, 3H, TiCH_3_), 0.46 (s, 3H, SiCH_3_) ppm. ^13^C{^1^H}-NMR (C_6_D_6_): δ 139.68, 139.38, 134.53, 134.17, 133.43, 114.31, 96.05, 53.84, 51.69, 51.49, 15.62, 15.24, 12.03, 1.05 ppm.

### 3.17. Compound ***18***

It was prepared by using the same procedure as employed for **9** by using **17**. A dark brown oil was obtained by removal of the solvent from the filtrate, which was pure based on ^1^H- and ^13^C-NMR analyses (1.26 g, 98%). ^1^H-NMR (C_6_D_6_): δ 4.23–4.10 (m, 2H, NCH_2_), 2.00 (s, 3H, CH_3_), 1.99 (s, 3H, CH_3_), 1.89 (s, 3H, CH_3_), 1.88 (s, 3H, CH_3_), 1.66 (q, *J =* 7.4 Hz, 2H, SiCH_2_), 1.37 (b, 2H, CH_2_), 1.09 (t, *J =* 7.4 Hz, 3H, CH_3_), 0.99 (t, *J =* 7.4 Hz, 3H, CH_3_), 0.49 (s, 3H, TiCH_3_), 0.48 (s, 3H, TiCH_3_), 0.37 (s, 3H, SiCH_3_) ppm. ^13^C{^1^H}-NMR (C_6_D_6_): δ 134.29, 133.87, 95.66, 53.41, 49.87, 28.48, 15.25, 14.79, 12.38, 12.03, 11.23, 6.97, 0.42 ppm.

### 3.18. Ethylene/1-Octene Copolymerization

Methylcyclohexane (25 mL) containing 1-octene (3.0 g) was introduced into a bomb reactor in a glove box. The reactor was assembled, removed out from the glove box, and heated to 130 °C using an oil bath. An activated catalyst was prepared by mixing the complex (0.5 μmol), iBu_3_Al (or [iBu_2_Al]_2_O) (0.20 mmol-Al), and [Ph_3_C][B(C_6_F_5_)_4_] (2.0 µmol) for 2 min. After adding the activated catalyst using a syringe, the system was immediately charged with ethylene gas (30 bar). The temperature increased rapidly due to the exothermic reaction, and the temperature profile is summarized in Table 1. The polymerization was conducted for 3 min using a continuous feed of ethylene at 30 bar and the reaction was quenched by venting the ethylene gas and introducing methanol through a syringe. After adding methanol (10 mL), the sticky polymer lump that was deposited was washed with methanol (10 mL) three times. The polymer was dried in a vacuum oven at 80 °C. The ^1^H-NMR spectra showed the methyl (CH_3_) signal (0.93–1.02 ppm) that was well isolated from the methine (CH) and methylene (CH_2_) signals (1.30–1.40 ppm), and the 1-octene content could be calculated by the signal intensities of the two regions. ^1^H-NMR spectra of the polymers (5 mg) were obtained at 70 °C after dissolution in deuterated toluene. It was proved that the 1-octene contents calculated from the ^1^H-NMR and ^13^C-NMR spectra are identical [26]. The polymer chain of the lowest molecular weight generated with CGC (*M*_n_, 9300; 1-octene content 21 mol %) is comprised of 41 1-octene units and 165 ethylene units, and the contribution of -CH_3_ end group to the intensity of the CH_3_ signal in the ^1^H-NMR spectrum is negligible.

## 4. Conclusions

RCM was successfully performed on ligand frameworks of CGC bearing allyl-Si (or vinyl-Si) and allyl-N units (Me_4_C_5_H-Si(Me)((CH_2_)_a_CH=CH_2_)-NH(C(R)(R′)CH=CH_2_), where R, R′ = H or Me; a = 0 or 1) to form six- or five-membered ring frameworks, -Si(Me_4_C_5_H)(Me)[CH_2_]_a_CH=CHC(R)(R′)NH-, by employing Schrock’s Mo-catalyst. Using the precursors and products of RCM, CGC derivatives [-Si(η^5^-Me_4_C_5_)(Me)CH_2_CH=CHC(R)(H)N-]TiMe_2_ (**13**, R = H; **15**, R = Me), [-Si(η^5^-Me_4_C_5_)(Me)CH_2_CH_2_CH_2_CH_2_N]TiMe_2_ (**14**), [(η^5^-Me_4_C_5_)Si(Me)(CH_2_CH=CH_2_)NCH_2_CH=CH_2_]TiMe_2_ (**16**), [(η^5^-Me_4_C_5_)Si(Me)(CH=CH_2_)NCH_2_CH=CH_2_]TiMe_2_ (**17**), and [(η^5^-Me_4_C_5_)Si(Me)(CH_2_CH_3_)NCH_2_CH_2_CH_3_]TiMe_2_ (**18**) were successfully prepared. The newly prepared complexes exhibit lower activity than the prototype CGC, [Me_2_Si(η^5^-Me_4_C_5_)(N*^t^*Bu)]TiMe_2_, when activated with [Ph_3_C][B(C_6_F_5_)_4_]/iBu_3_Al, but the catalytic activity of these species was significantly improved by using [iBu_2_Al]_2_O instead of iBu_3_Al. The **14/**[Ph_3_C][B(C_6_F_5_)_4_]/[iBu_2_Al]_2_O and **18/**[Ph_3_C][B(C_6_F_5_)_4_]/[iBu_2_Al]_2_O species exhibit high activity, comparable to that of CGC/[Ph_3_C][B(C_6_F_5_)_4_]/iBu_3_Al (4.7, 3.5, and 5.0 × 10^6^ g/mol-Ti, respectively). Advantageously, higher molecular weight polymers were produced with narrow molecular weight distributions by using **14** and **18** (*M*_n_, 36, 69, and 9.3 kDa for **14**, **18** and CGC, respectively).

## Figures and Tables

**Table 1:** Ethylene/1-octene copolymerization results ^a^.

Entry	Cat	Scavenger	Temperature (°C)	Yield (g)	Activity ^b^	[Oct] ^c^ (mol %)	*M*_n_ (kDa)	*M*_w_/*M*_n_
1	**13**	iBu_3_Al	129-139-134	0.91	1.82	12	69.4	2.02
2	**14**	iBu_3_Al	130-142-135	0.89	1.78	14	50.4	2.14
3	**15**	iBu_3_Al	129-147 (50 sec)-137	1.54	3.08	11	43.0	2.17
4	**16**	iBu_3_Al	129-132-129	0.42	0.84	14	108	2.18
5	**17**	iBu_3_Al	129-136-135	0.61	1.22	15	105	2.30
6	**18**	iBu_3_Al	129-144 (40 sec)-137	1.19	2.38	19	72.4	1.97
7	CGC	iBu_3_Al	120-143 (25 sec)-125	2.48	4.96	21	17.0	4.41
8	**13**	[iBu_2_Al]_2_O	129-148-142	1.53	3.06	14	44.8	2.17
9	**14**	[iBu_2_Al]_2_O	128-153 (40 sec)-146	2.36	4.72	15	35.6	2.27
10	**14**	[iBu_2_Al]_2_O	80-112 (60 sec)-113	3.28	6.55	15	203	2.16
11	**15**	[iBu_2_Al]_2_O	129-154-150	2.75	5.50	13	26.1	2.22
12	**16**	[iBu_2_Al]_2_O	128-136-133	0.90	1.80	14	107	2.31
13	**17**	[iBu_2_Al]_2_O	129-137-133	1.01	2.02	16	105	2.17
14	**18**	[iBu_2_Al]_2_O	128-147 (50 sec)-138	1.73	3.46	20	68.8	1.90
15	CGC	[iBu_2_Al]_2_O	120-154 (50 sec)-134	3.58	7.15	21	9.3	6.87
16	CGC	[iBu_2_Al]_2_O	80-121 (35 sec)-108	4.20	8.40	36	33.7	1.91

^a^ Polymerization conditions: methylcyclohexane solution (25 mL) containing 1-octene (3.0 g), 0.5 µmol Ti, 2.0 µmol [Ph_3_C][B(C_6_F_5_)_4_], scavenger (0.20 mmol-Al), ethylene (30 bar), 3 min. ^b^ Activity in unit of 10^6^ g/mol-Ti. ^c^ 1-Octene content in the copolymer determined by ^1^H-NMR.
